# Rurality and COVID-19 Outcomes: Unraveling the Impact of Nursing Home Residency Using Bayesian Analysis

**DOI:** 10.3390/jcm13237244

**Published:** 2024-11-28

**Authors:** Javier Martínez-Redondo, Montserrat Crespo Pons, Alicia Mateu Llevadot, Jesús Pujol Salud, Carles Comas

**Affiliations:** 1 Primary Health Care Area of Balaguer, Institut Català de la Salut [ICS], 25600 Lleida, Spain; jmartinez.lleida.ics@gencat.cat (J.M.-R.); mcrespo.lleida.ics@gencat.cat (M.C.P.); amateu.lleida.ics@gencat.cat (A.M.L.); 2 Instituto de Investigación Biomédica [IRB Lleida], Universidad de Lleida [UdL], 25198 Lleida, Spain; 3 Departamento de Matemáticas, Campus Escola Tècnica Superior d’Enginyeria Agroalimentària i Forestal i de Veterinària, Universidad de Lleida, 25001 Lleida, Spain; carles.comas@udl.cat

**Keywords:** rurality, COVID-19, nursing homes, Bayesian analysis

## Abstract

**Background and Objectives**: Many studies have analyzed the impact of rurality on the incidence and consequences of COVID-19 infection. However, these studies have not considered the impact of different numbers of nursing homes in rural, semi-urban, or urban areas. Our objective was to analyze the effect of the factor of rurality on the incidence and mortality of COVID-19 while accounting for the impact of the variable of nursing home residency. In addition, we performed a comparative analysis of the infected population in semi-urban and rural areas. **Methods**: We first analyzed COVID-19 infection in all populations in the Balaguer Primary Health Care Area before examining the impact of rurality using Bayesian logistic regression analysis, specifically excluding the population living in nursing homes. We also performed an epidemiological and clinical analysis comparing rural and semi-urban areas. **Results**: We found higher incidence of and higher relative and absolute mortality from COVID-19 infection in semi-urban areas than in rural areas. After excluding nursing home residents from our sample, the Bayesian analysis indicated that rurality was not protective against COVID-19 infection or mortality. The incidence rates, specific mortality rates, and case fatality rates were similar in semi-urban and rural areas. All comorbidities, except chronic obstructive pulmonary disease, were associated with higher mortality, while no symptoms were associated with higher mortality. **Conclusions**: Excluding the population residing in nursing homes from the analysis, we found that rurality was not a protective factor against either infection or mortality during the first COVID-19 wave. Our Bayesian model analysis confirmed that rurality alone did not enhance survival among residents of rural areas.

## 1. Introduction

One key question in scientific research on the COVID-19 pandemic is how geographical dispersion affected the epidemics. Specifically, such a question referred to whether higher or lower population density influenced the consequences of the pandemic [1,2,3,4,5].

Some studies indicated that higher population density caused higher COVID-19 mortality [3,4,6,7]. Other studies found higher mortality rates in non-metropolitan areas [8,9,10], and others, a higher prevalence of cumulative COVID-19 cases in metropolitan areas than in non-metropolitan areas until the end of October 2020, and after this, the prevalence of cumulative COVID-19 cases was higher in non-metropolitan areas, although as from April 2021, the prevalence of cumulative COVID-19 cases has been similar in metropolitan and non-metropolitan areas [11].

Our literature review is based on international reports based on studies of populations varying in social and economic characteristics and with very different health care systems, particularly studies conducted in the United States. Those differences could explain the contradictory results on the links between rurality and COVID-19-related risks.

The different results among published studies could also be explained by work-related mobility, as some rural communities rely on industrial and urban jobs. Urban jobs also entail high mobility and therefore expose the workers of such jobs to infectious diseases through mass transportation and by placing them in crowded urban areas.

However, we must consider the significant impact of the pandemic on nursing homes, which attracted considerable epidemiologic interest regarding incidence and mortality rates [12,13,14] during the first wave of COVID-19 infection, from March to June 2020 [15]. Therefore, the varying number of nursing homes in urban versus rural areas could influence the results of studies addressing whether rurality serves as a protective factor against COVID-19 infection and/or mortality.

After an extensive literature review, we found no studies that have analyzed the rurality factor on COVID-19 incidence and mortality while considering the impact of the variable of “nursing home residency”.

Moreover, although the existing literature highlights the important role of primary care [16,17,18,19] in implementing immediate prevention, early detection, and control measures for patients with COVID-19, we have only found one study carried out by primary care services, in Spain, where the rurality factor in the context of COVID-19 infection was evaluated [20]. However, this study did not analyze the variable of patients admitted to nursing homes under the scope of the rurality factor.

Our research group aimed to analyze the impact of the rurality factor on COVID-19 incidence and mortality, while considering the variable of nursing home residency, during the first wave of COVID-19 infection. Specifically, a retrospective observational study was carried out in which we performed a comparative analysis between the infected population in the semi-urban Balaguer municipality, which has 16,841 inhabitants, and the infected population in the rural area of the surrounding municipalities with fewer than 2500 inhabitants, all served by the same primary care center.

The Balaguer Primary Care Center is the health center that serves a reference area in the Noguera region in the province of Lérida in the region of Catalonia in Spain. Spain has a National Health System with universal access financed by the national budget and managed by each region. The region of Catalonia manages its health budget and is responsible for organizing and providing services. Primary health care and hospital care are publicly funded, including medical medications, which are mostly paid for with public funds for people with chronic diseases and people over 65 years of age.

## 2. Materials and Methods

### 2.1. Study Design and Participants

This retrospective observational study was conducted in the Balaguer Primary Health Care Area (PHC area) from 13 March to 28 May 2020. Inclusion criteria were being over 20-years-old (adult population) and being actively listed in the patient census assigned to the PHC area.

### 2.2. Sample

In March 2020, the total population assigned to the Balaguer PHC area was 28,047 individuals, of whom 22,458 were over 20-years-old. Among these, 13,570 lived in the Balaguer municipality (population density 306.1 inhabitants/km^2^), while 8888 lived in population centers with fewer than 2500 inhabitants (population density 15.72 inhabitants/km^2^). These were defined as our two population groups. For readability, we will refer to the Balaguer municipality as the semi-urban area and to its surrounding municipalities with fewer than 2500 inhabitants as the rural area throughout the text (as specified in other studies [2,21]).

The Balaguer PHC area covers 6 nursing homes with a total of 406 beds; the semi-urban area has 282 nursing home beds (representing 2.08% of its population), while its surrounding rural area has 124 beds (representing 1.39% of their population).

To determine the impact of the rurality variable on COVID-19 infection, we first examined some differentiating factors that could influence our findings, such as climate, per capita income, and the prevalence of nursing home residency. Both the semi-urban area and the rural area, served by the Balaguer PHC area, have similar climatic characteristics [22] and a similar per capita income of approximately 24,000 € [23].

### 2.3. Variables

The following variables were collected in the database: age, sex, residency in a nursing home or not, residency in the semi-urban or rural area, clinical evolution during the month following infection or symptom onset (recovered/deceased), hospitalization, fever over 37 °C, sore throat, sputum production, cough, headache, shortness of breath, loss of smell, fatigue, and diarrhea. Comorbidities assessed included obesity, hypertension, diabetes mellitus, chronic obstructive pulmonary disease (COPD), cardiovascular disease, chronic kidney disease, history of cancer, and neurological disorders.

### 2.4. Data Collection

Due to the global evolution of the COVID-19 pandemic, especially in Spain (as of 13 March 2020, with 3869 infected and 90 deceased), the management of the Balaguer PHC area established a respiratory isolation unit (COVID-19 Unit) on 13 March. This unit, located at the Balaguer Primary Care Center, operated 24 h a day to care for individuals suspected or confirmed to have COVID-19. Given the epidemiological urgency and the severity of the pandemic, along with the lack of information available in February and March 2020, a database was created on 16 March 2020. This database aimed to track the clinical evolution of patients, study symptoms, evaluate comorbidities associated with COVID-19 infection, and improve population-level clinical decision-making (such as identifying initial cases for contact tracing, establishing local health policies, etc.) and individual clinical decisions (improving early diagnosis, implementing treatments, studying referrals, etc.).

Every 48 h, the COVID-19 Unit reviewed the information on all deceased patients within the Balaguer PHC area and collected information on all new COVID-19 cases, generating a single database with the daily cumulative incidence along with various clinical and population variables. This database included patients treated within the COVID-19 Unit, those receiving continuous care (from 8:00 P.M. to 8:00 A.M.), and patients visited in nursing homes.

This database of confirmed cases included all patients with a confirmed diagnosis of COVID-19 infection in the Balaguer PHC area. It also included deceased patients who lacked RT-PCR testing but whose clinical histories suggested COVID-19 infection as the probable cause of death. The presumed cause of death from COVID-19 was determined based on a lack of response to antibiotics and all clinical and epidemiological data pointing in that direction (e.g., close contact with other infected patients in a nursing home or family members), with no alternative causes identified before symptom onset.

### 2.5. Statistical Analysis

We calculated both absolute and relative frequencies of the study variables across the two population groups. Additionally, we calculated the incidence rate (number of cases per exposed population per 1000 inhabitants), the specific mortality rate (COVID-19-related deaths per total population per 1000 inhabitants), and the case fatality rate (COVID-19-related deaths per infected patients per 100 inhabitants) for the overall sample and within each population group.

We created contingency tables to compare different variables and to evaluate COVID-19-related mortality in relation to our variables of interest. We performed a chi-square test to evaluate statistical differences between the two population groups, considering *p* < 0.05 as statistically significant. We also analyzed the linear dependence between age and different comorbidities using Pearson’s correlation coefficient. All statistical analyses were performed using the R statistical package, version 4.0.2 (R Core Team, Vienna, Austria, 2020).

We applied a Bayesian logistic regression model to analyze the rurality variable. To examine a response variable, *y*, that can only take two values—in our dataset, survival or death of an individual, coded as 1 or 0—we considered a (binary) logistic regression model, Gelman and Hill, 2007 [24], *y_i_ ~ Bernoulli*[*p_i_*], where *i* = 1, …, *n* individuals. Typically, this model is defined as

$$\ln\left(\frac{p_{i}}{\left(1-p_{i}\right)}\right)=\hat{\alpha }+\hat{\beta }\;x_{i}$$

where $x_{i}$ is a vector of explanatory variables, $\hat{\alpha }$ is the intercept, $\hat{\beta }$ is a vector of parameters, and $p_{i}$ is the probability of success for the individual i (in this case, the probability of the individual survival). In our study, we used “non-informative” priors for all parameters, meaning a normal distribution with zero mean and a large variance because we had little prior knowledge about the parameters to be estimated and we assumed that these values can be far from zero. Bayesian estimation was performed via Markov Chain Monte Carlo (MCMC) simulation using the RSTANARM package of the R project, Goodrich et al., 2020 [25].

We applied a leave-one-out (LOO) cross-validation approach to measure the predictive fit of the different models analyzed. LOO cross-validation estimates the expected log predictive density (ELPD) for a new dataset, similar to the Akaike Information Criterion (AIC) used in non-Bayesian methods. In this study, we present both measures—the LOO-ELPD and the AIC—to evaluate the performance of the analyzed models.

We used a credible interval (95% CrI) for all evaluations of the model coefficients, using the posterior coefficient distribution derived from 4000 simulations.

## 3. Results

### 3.1. Prior Analysis of the Variable “Nursing Home Residency” and Patient Characteristics

During the study period, a total of 211 patients were diagnosed with COVID-19: 144 resided in the semi-urban area, with 81 (56.25%) living in nursing homes, and 67 lived in the rural area, of whom 27 (40.29%) were nursing home residents.

Our preliminary analysis also showed significant differences (X^2^ = 5.4505, *p* = 0.01956) between the groups by examining the variable incidence and rurality together. Specifically, a higher incidence rate and higher relative and absolute mortality were found in the population over 20-years-old in the semi-urban area (Table 1). However, some data analyzed (see in Table 1) led us to think that the higher concentration of nursing homes in the semi-urban area versus the rural area could introduce significant bias in the analysis [14].

Of the 211 patients diagnosed, 108 (51.18%) lived in nursing homes, and 103 (48.81%) lived in the community. COVID-19 incidence in the nursing homes in the semi-rural area was 29% (61 times higher than in the population who lived in the semi-rural area outside nursing homes), while it was 22% in nursing homes in the rural area (48 times higher than in the population who lived in the rural area outside nursing homes).

In the total infected population, mortality occurred mainly in nursing homes. A total of 41 (80.39%) patients who died in nursing homes; while 10 (19.60%) patients who died lived outside nursing homes. The case fatality rate was 38% in nursing homes, compared to 9.5% among the remaining population older than 20 dwelling in the Balaguer PHC area.

The specific mortality rate (SMR) from COVID-19 was also higher in the nursing homes (101‰) than in the population residing outside nursing homes (0.45‰).

The total of the infected population that was admitted to a hospital was 51 patients (24.17%) (32 from the semi-rural area and 9 from the rural area), and of these 18 died (13 from the semi-rural area and 9 from the rural area).

Therefore, to avoid this potential bias, we were forced to exclude from the analysis all patients living in nursing homes, resulting in a final sample of 103 patients infected with COVID-19. Among them, 63 patients (61.16%) resided in the semi-urban area, with a mean age of 51 years, of whom 26 (41.27%) were men. The remaining 40 patients (38.83%) lived in the rural area, with a mean age of 58 years, of whom 18 (45%) were men.

COVID-19 infection was confirmed by RT-PCR in 101 patients (98%), while 2 patients (2%) died with suspected infection.

### 3.2. The Correlation of the Rurality Variable with COVID-19 Mortality and Incidence

The incidence of COVID-19 infection was 4.74‰ in the semi-urban area and 4.56‰ in the rural area (Table 2).

Out of the 103 infected patients, 10 died: 6 residing in the semi-urban area and 4 living in the rural area. The specific mortality rates were 0.451‰ in the semi-urban area and 0.456‰ in the rural area, while the case fatality rates were 9.52% in the semi-urban area and 10% in the rural area (Table 2).

We found no significant differences between COVID-19 mortality according to the patient’s residency—patients residing in the semi-urban area or rural area (X^2^ = 0.0063, *p* = 0.9366). Similarly, incidence rates did not differ significantly (X^2^ = 0.0355, *p* = 0.8504) in the total sample (*n* = 22,052) (Table 2).

The analysis of symptoms, obesity, comorbidities, and hospital admissions between the infected population of the semi-urban area and rural area showed great homogeneity. We only observed statistically significant differences in the presence of headaches and obesity (Table 3). These results show that the health status of the rural population is very similar to that of the semi-rural population; for this reason, we do not consider it necessary to control for these factors in the analysis of semi-rural–rural differences in the incidence or mortality of COVID. 

### 3.3. Correlation of Symptoms and Comorbidities with COVID-19 Mortality

We also explored the relationships between symptoms, comorbidities, and mortality (Table 4).

All comorbidities except COPD were associated with higher mortality, while no specific symptoms were associated with higher mortality.

### 3.4. Correlation of Age and Comorbidities

We also investigated the relationship between patient age and comorbidities, as some conditions might be expected to worsen with age. We did not analyze the relationship between age and symptoms, as symptoms did not appear to be relevant predictors of COVID-19 mortality in our dataset. Table 5 shows the Pearson correlation coefficient between age and the mean number of each comorbidity within five-year age groups. Except for neoplasia and neurological pathologies (correlation ro = 0.1764, *p* = 0.0746), we found a significant positive correlation between age and the presence of comorbidities, indicating that as patients age, their comorbidities tend to increase. Furthermore, our analysis of rurality revealed that age does not significantly correlate with residing in rural or semi-urban areas (ro = 0.1916, *p* = 0.0524).

### 3.5. Age and/or Rurality as Predictors of COVID-19 Mortality

We subsequently applied several Bayesian logistic regression models to analyze whether rurality and/or age could predict patient survival.

The first model we analyzed aimed to predict COVID-19 mortality based solely on age (outcome as death or recovery according to age). The 95% credible interval (CrI) for the age predictor does not include the value zero and contains negative values, indicating that older age is directly associated with lower survival rates (Table 6).

We included the rurality variable in two models: one where rurality was the sole predictor of mortality (outcome as death or survival based on rurality) and another where age was also incorporated (outcome as death or survival based on age and rurality). Both models performed better than the age-only model in terms of predictive accuracy (Table 6). However, despite the better predictive performance indicated by the ELPDOO and AIC values, rurality was not a strong predictor of survival after COVID-19 infection. The 95% CrI for rurality in both models included the value zero, indicating that rurality is not a reliable factor in predicting COVID-19 survival. These findings suggest that living in rural areas did not serve as a protective factor against COVID-19 mortality.

## 4. Discussion

Some studies have shown that urban or semi-urban areas experienced more severe COVID-19 impacts than rural regions [4,6,7]. Others showed higher infection rates in rural populations [8,9,10]. However, none of these studies accounted for the variable of nursing home residency.

In the initial analysis of our study, the semi-urban area, with a greater number of residents in nursing homes, had a higher incidence of COVID-19 infection and higher mortality by COVID-19 than in the rural area served by the same health services and with similar climate and income characteristics. By excluding the population residing in nursing homes from the analysis, we found that rurality was not a protective factor against either infection or mortality during the first COVID-19 wave. Our Bayesian model analysis confirmed that rurality alone did not enhance survival among residents of the rural area.

In our symptom analysis, like others, the only significant difference was a higher frequency of headaches in the rural population. Notably, no specific symptoms were associated with higher mortality [16,17,26,27]. In our comorbidity analysis, we found no significant differences between semi-urban and rural populations. Like other studies [16,17,26,27,28], all comorbidities, except chronic obstructive pulmonary disease, were associated with increased mortality. We also found no significant differences in hospitalization rates between rural and semi-urban populations, although hospitalization was associated with higher mortality. We analyzed the correlation between comorbidities, rurality, and age. All comorbidities, except for neoplasia and neurological conditions, were associated with older age; however, this correlation did not extend to rurality.

Our study is the first one to evaluate rurality as a protective factor against COVID-19, excluding nursing home residents, using a retrospective database of patients from rural and semi-urban areas during the first wave of COVID-19. Our extensive database allowed for a detailed statistical analysis of rurality using innovative Bayesian methods.

The main limitation of the study is its external validity given the specific characteristics of the study population as explained in the context above. Internal validity is good since both populations have access to the same primary health care and hospital care system. Another limitation of our study is that we have not analyzed the impact of population mobility from one area to another for reasons of work, leisure, etc. However, we consider that the impact of mobility would not modify our results because our study was carried out during the first wave of the infection when the strong limitations on mobility imposed by the Spanish government were in force. Another limitation of our study is the sample size, although it reflects events during the first wave of COVID-19 in a high-incidence area, with more than 28,000 inhabitants. Another limitation of our study is the lack of RT-PCR testing during this period complicated the analysis of mortality associated with COVID-19 as a dependent variable and may have led to an overestimation of the case fatality rate and an underestimation of the incidence rate. Despite this, we believe that our study is valid because the lack of RT-PCR tests equally affected both groups studied and in any case does not affect the main objective of our study.

## 5. Conclusions

This study contributes to a clearer understanding of the first wave of COVID-19 in rural and semi-urban areas, which can inform future public health decision-making regarding infectious diseases. This includes guiding the allocation of booster vaccine doses and new treatments, without relying solely on urban density as a determining factor. Additionally, our findings suggest the need for implementing similar contagion-control policies in both rural and semi-urban areas. Moreover, we cannot forget the great impact of the pandemic on the population admitted to nursing homes. The results of this study should lead us to prioritize public health measures (prioritization of vaccination, administration of new drugs, provision of detection mechanisms, etc.) for patients admitted to nursing homes.

## Figures and Tables

**Table 1:** The impact of the variable “nursing home residency” on the correlation of the rurality variable with the COVID-19 infection status.

	Semi-Urban Area	Rural Area
Total population > 20-years-old (*n* = 22,458)	13,570 (60.4%)	8888 (39.6%)
Total population > 20-years-old residing in nursing homes served by the Balaguer PHC area (*n* = 406)	282 (2.08%)	124 (1.39%)
Total population > 20-years-old not residing in nursing homes (*n* = 22,052)	13,288 (60.26%)	8764 (39.74%)
Total population > 20-years-old infected (*n* = 211)	144 (1.06%)	67 (0.75%)
Total of the infected population who died (*n* = 51)	42 (82.35%)	9 (17.63%)
Population residing in nursing homes infected (*n* = 108)	81 (75%)	27 (25%)
Population not residing in nursing homes with infection (*n* = 103)	63 (0.47%)	40 (0.46%)
Population residing in nursing homes infected who died (*n* = 41)	36 (87.8%)	5 (12.2%)
Population not residing in nursing homes infected who died (*n* = 10)	6 (60%)	4 (40%)
Case fatality rate in nursing home patients per 100 inhabitants	33.4%	4.6%
Case fatality rate in patients not residing in nursing homes per 100 inhabitants	5.82%	3.88
Total of the infected population admitted to the hospital (*n* = 51)	32 (62.74%)	19 (37.26%)
Total of the infected population admitted to the hospital who died (*n* = 18)	13 (72.22%)	5 (27.78%)
Incidence in nursing homes	29%	22%
Incidence in the population over 20-years-old per 1000 inhabitants (*n* = 22,458)	10.61‰	7.53‰
“X^2^” Infection-rurality in the total population (*n* = 22,458)	X^2^ = 5.4505 (*p* = 0.01956)	

**Table 2:** Incidence, mortality rate, and case fatality rate.

	Semi-Urban Area	Rural Area
Incidence per 1000 inhabitants	4.74‰	4.56‰
Specific mortality rate per 1000 inhabitants	0.451‰	0.456‰
Case fatality rate per 100 inhabitants	9.52%	10%
Association between mortality and residency	X^2^ = 0.0063 (*p* = 0.9366)	
Association between being infected and residency	X^2^ = 0.0355 (*p* = 0.8504)	

**Table 3:** A comparative analysis of symptoms and comorbidities between the two population groups.

Symptoms, Comorbidities, and Hospital Admission	Total Patients *n* = 103	Semi-Urban Area *n* = 63	Rural Area *n* = 40	Chi-Square
Fever	67 (65.04%)	39 (61.9%)	28 (70%)	0.7052 (*p* = 0.401)
Odynophagia	5 (4.85%)	3 (4.76%)	2 (5%)	0.0030 (*p* = 0.956)
Expectoration	1 (0.97%)	0 (0%)	1 (2.5%)	3.2123 (*p* = 0.073)
Cough	47 (45.63%)	29 (46.03%)	18 (45%)	0.0105 (*p* = 0.918)
Headache	14 (13.59%)	4 (6.34%)	10 (25%)	**7.2462 (*p* = 0.007)**
Dyspnea	29 (28.15%)	19 (30.15%)	10 (25%)	0.3218 (*p* = 0.570)
Anosmia	15 (14.56%)	9 (14.28%)	6 (15%)	0.0100 (*p* = 0.920)
Asthenia	31 (30.09%)	21 (33.33%)	10 (25%)	0.8075 (*p* = 0.368)
Diarrhea	10 (9.70%)	5 (7.93%)	5 (12.5%)	0.5812 (*p* = 0.445)
Obesity	22 (21.35%)	8 (12.69%)	14 (35%)	**7.2443 (*p* = 0.007)**
Arterial hypertension	29 (28.15%)	16 (25.39%)	13 (32.5%)	0.6102 (*p* = 0.434)
Diabetes	14 (13.59%)	8 (12.69%)	6 (15%)	0.1103 (*p* = 0.739)
COPD	12 (11.65%)	8 (12.69%)	4 (10%)	0.1730 (*p* = 0.677)
Heart disease	16 (15.53%)	9 (14.28%)	7 (17.5%)	0.1926 (*p* = 0.660)
Chronic renal failure	6 (5.82%)	3 (4.76%)	3 (7.5%)	0.3343 (*p* = 0.563)
Neoplasm’s history	5 (4.85%)	3 (4.76%)	2 (5%)	0.0030 (*p* = 0.956)
Neurological pathology	5 (4.85%)	2 (3.17%)	3 (7.5%)	0.9910 (*p* = 0.319)
Hospital admission	32 (31.06%)	17 (16.5%)	15 (14.56%)	1.2633 (*p* = 0.261)

Bold remarks statistical significance.

**Table 4:** The analysis of potential associations between symptoms, comorbidities, and mortality in the overall sample.

Symptoms, Comorbidities, and Hospital Admission	Total Patients *n* = 103	Mortality	Survival	Chi-Square
Fever	67	9 (13.43%)	58 (86.57%)	3.0328 (*p* = 0.081)
Odynophagia	5	1 (20%)	4 (80%)	0.6349 (*p* = 0.425)
Expectoration	1	0 (0%)	1 (100%)	0.1085 (*p* = 0.741)
Cough	47	3 (6.38%)	44 (93.62%)	1.0907 (*p* = 0.296)
Headache	14	2 (14.29%)	12 (85.71%)	0.3871 (*p* = 0.533)
Dyspnea	29	5 (17.24%)	24 (82.76%)	2.6127 (*p* = 0.106)
Anosmia	15	1 (6.66%)	14 (93.44%)	20.1853 (*p* = 0.666)
Asthenia	31	3 (9.68%)	28 (90.32%)	0.0000 (*p* = 0.994)
Diarrhea	10	2 (20%)	8 (80%)	1.3381 (*p* = 0.247)
Obesity	22	4 (18.18%)	18 (81.82%)	2.2911 (*p* = 0.130)
Arterial hypertension	29	7 (24.14%)	22 (75.86%)	**9.5869 (*p* = 0.002)**
Diabetes	14	6 (42.85%)	8 (57.15%)	**20.3092 (*p* = 0.000)**
COPD	12	3 (25%)	9 (75%)	3.6229 (*p* = 0.057)
Heart disease	16	5 (31.25%)	11 (68.75%)	**10.027 (*p* = 0.001)**
Chronic renal failure	6	3 (50%)	3 (50%)	**11.799 (*p* = 0.000)**
Neoplasm’s history	5	3 (60%)	2 (40%)	**15.162 (*p* = 0.000)**
Neurological pathology	5	2 (40%)	3 (60%)	**5.5006 (*p* = 0.019)**
Hospital admission	32	8 (25%)	24 (75%)	**12.382 (*p* = 0.000)**

Bold remarks statistical significance.

**Table 5:** The Pearson correlation coefficient between patient age and the mean number of observations of each comorbidity within 5-year age groups.

Comorbidities	Pearson Correlation Coefficient—ρ (ro)	*p*-Value
Chronic renal failure	0.391	0.0000
Diabetes	0.379	0.0000
Arterial hypertension	0.602	0.0000
Chronic obstructive pulmonary disease	0.266	0.0064
Heart disease	0.416	0.0000
Neoplasia	0.176	0.0746
Neurological pathology	0.183	0.0631
Rurality	0.191	0.0524

**Table 6:** Bayesian logistic regression models. Mortality outcome: age vs. rurality vs. age and rurality.

Model	α (CrI)	β_1_ (CrI)	β_2_ (CrI)	ELPD-LOO	AIC
Mortality outcome—Age	9.826 (6.000, 14.537)	−0.115 (−0.178, −0.062)		−22.1	45
Mortality outcome—Rurality	2.261 (1.543, 3.232)	**−0.046** **(−1.375, 1.336)**		−34.9	70
Mortality outcome—Age-Rurality	9.657 (5.853, 15.088)	−0.115 (−0.185, −0.063)	**0.476** **(−1.096, 2.219)**	−23.1	46

Bold remarks statistical significance.

## Data Availability

All study data is presented in this article, either directly or indirectly.
